# Predictors of Death or Severe Impairment in Neonates With Hypoxic-Ischemic Encephalopathy

**DOI:** 10.1001/jamanetworkopen.2024.49188

**Published:** 2024-12-05

**Authors:** Hannah C. Glass, Thomas R. Wood, Bryan A. Comstock, Adam L. Numis, Sonia L. Bonifacio, Marie-Coralie Cornet, Fernando F. Gonzalez, Adriana Morell, Sarah E. Kolnik, Yi Li, Amit Mathur, Ulrike Mietzsch, Tai-Wei Wu, Courtney J. Wusthoff, Marianne Thoresen, Patrick J. Heagerty, Sandra E. Juul, Yvonne W. Wu

**Affiliations:** 1 Department of Neurology, Weill Institute for Neuroscience, University of California, San Francisco; 2 Department of Pediatrics, UCSF Benioff Children’s Hospital, University of California, San Francisco; 3 Department of Epidemiology & Biostatistics; University of California, San Francisco; 4 Department of Pediatrics, Division of Neonatology, University of Washington School of Medicine, Seattle Children’s Hospital, Seattle; 5 Department Biostatistics, University of Washington, Seattle; 6 Department of Pediatrics, Division of Neonatal and Developmental Medicine, Stanford University, Palo Alto, California; 7 Department of Radiology and Biomedical Imaging, University of California, San Francisco; 8 Department of Pediatrics, St Louis University School of Medicine, St Louis, Missouri; 9 Fetal and Neonatal Institute, Children’s Hospital Los Angeles, Keck School of Medicine of USC, Los Angeles, California; 10 Department of Neurology, Stanford University, Palo Alto, California; 11 Neonatal Neuroscience, Translational Health Sciences, University of Bristol, Bristol, United Kingdom; 12 Section for Physiology, Institute of Basic Medical Sciences, University of Oslo, Oslo, Norway

## Abstract

**Importance:**

Outcomes after hypoxic-ischemic encephalopathy (HIE) are variable. Predicting death or severe neurodevelopmental impairment (NDI) in affected neonates is crucial for guiding management and parent communication.

**Objective:**

To predict death or severe NDI in neonates who receive hypothermia for HIE.

**Design, Setting, and Participants:**

This prognostic study included participants enrolled in a large US clinical trial conducted in US neonatal intensive care units who were born between January 2017 and October 2019 and followed up to age 2 years. Eligible participants were neonates with moderate-severe HIE born at 36 weeks or more gestation and with 2-year outcome data. Data were analyzed June 2023. External validation was performed with a UK cohort.

**Exposure:**

Clinical, electroencephalography (EEG), and magnetic resonance imaging (MRI) variables were curated and examined at 24 hours and following cooling.

**Main Outcome and Measures:**

Death or severe NDI at age 2 years. Severe NDI was defined as Bayley Scales of Infant Toddler Development cognitive score below 70, Gross Motor Function Classification System score of 3 or higher, or quadriparesis. Model performance metrics were derived from training, internal, and external validation datasets.

**Results:**

Among 424 neonates (mean [SD] gestational age, 39.1 [1.4] weeks; 192 female [45.3%]; 28 Asian [6.6%], 50 Black [11.8%], 311 White [73.3%]), 105 (24.7%) had severe encephalopathy at enrollment. Overall, 59 (13.9%) died and 46 (10.8%) had severe NDI. In the 24-hour model, the combined presence of 3 clinical characteristics—(1) severely abnormal EEG, (2) pH level of 7.11 or below, and (3) 5-minute Apgar score of 0—had a specificity of 99.6% (95% CI, 97.5%-100%) and a positive predictive value (PPV) of 95.2% (95% CI, 73.2%-99.3%). Validation model metrics were 97.9% (95% CI, 92.7%-99.8%) for internal specificity, with a PPV of 77.8% (95% CI, 43.4%-94.1%), and 97.6% (95% CI, 95.1%-99.0%) for external specificity, with a PPV of 46.2% (95% CI, 23.3%-70.8%). In the postcooling model, specificity for T1, T2, or diffusion-weighted imaging (DWI) abnormality in at least 2 of 3 deep gray regions (ie, thalamus, caudate, putamen and/or globus pallidus) plus a severely abnormal EEG within the first 24 hours was 99.1% (95% CI, 96.8%-99.9%), with a PPV of 91.7% (95% CI, 72.8%-97.8%). Internal specificity in this model was 98.9% (95% CI, 94.1%-100%), with a PPV of 92.9% (95% CI, 64.2%-99.0%); external specificity was 98.6% (95% CI, 96.5%-99.6%), with a PPV of 83.3% (95% CI, 64.1%-93.4%).

**Conclusions and Relevance:**

In this prognostic study of neonates with moderate or severe HIE who were treated with therapeutic hypothermia, simple models using readily available clinical, EEG, and MRI results during the hospital admission had high specificity and PPV for death or severe NDI at age 2 years.

## Key Points

**Question:**

What clinical factors predict death or severe neurodevelopmental impairment (NDI) among infants treated with hypothermia for hypoxic-ischemic encephalopathy (HIE)?

**Findings:**

This prognostic study including 424 neonates and using recursive binary partitioning identified key factors within 24 hours after birth (including severely abnormal electroencephalogram [EEG], pH of 7.11 or below, and 5-minute Apgar score of 0) and after cooling (2 of 3 deep gray regions injured on magnetic resonance imaging [MRI] and severely abnormal 24-hour EEG) that, when present, were specific and had high positive predictive value for death or severe NDI in internal and external validation data.

**Meaning:**

These results suggest that among infants with HIE clinical, EEG and MRI have high specificity to predict death or severe NDI at age 2 years.

## Teaser

This prognostic study of neonates with moderate-severe hypoxic-ischemic encephalopathy enrolled in a large US clinical trial examines clinical characteristics that may predict death or severe neurodevelopmental impairment.

## Introduction

Perinatal hypoxic-ischemic encephalopathy (HIE) occurs in approximately 1.5 per 1000 live births and is a leading cause of morbidity and mortality in the US and worldwide.^1^ Outcome is variable, with nearly half of infants having normal neurodevelopment at age 2 years, and the remainder sustaining a spectrum of neurodevelopmental impairment (NDI) or early death. Timely and accurate prediction of death or severe NDI in affected neonates is crucial for guiding management and communication.

Existing tools to predict outcome in neonates with HIE are limited by single-center studies, small study size, and limited types of predictors (eg, using biomarkers or imaging techniques that are not widely available,^2,3,4,5^ or using only clinical,^6,7,8,9,10,11,12,13,14^ neurophysiology,^15,16^ or neuroimaging results,^17,18,19,20,21^ or combining 2 of the 3^22,23,24,25,26^). A 2023 systematic review^27^ concluded that existing models are of insufficient quality to be incorporated into clinical practice.

To overcome limitations of prior studies, we used data from the High-Dose Erythropoietin for Asphyxia and Encephalopathy (HEAL) trial (ClinicalTrials.org identifier, NCT02811263),^28^ and a prospective, population-based cohort of neonates with encephalopathy from the UK to train, test, and validate models. The HEAL trial was a randomized, double-masked trial of erythropoeitin (5 doses of 1000 IU per kilogram of body weight administered intravenously over the first week after birth) compared with placebo that showed no neuroprotective effect in 500 neonates with moderate to severe HIE. We hypothesized that readily available data from clinical, neurophysiology, and magnetic resonance imaging (MRI) reports could be used to develop a decision tree to predict neonates with at least 85% likelihood of death or severe NDI.

## Methods

### Overview

The HEAL protocol^29^ and primary results of 500 infants born January 2017 to October 2019 with moderate to severe HIE who received therapeutic hypothermia were previously published.^28^ This study was approved by the institutional review boards at all sites and participants were enrolled after written informed consent obtained prenatally. The approach toward building and reporting clinical prediction models adhered to the most recent Transparent Reporting of a multivariable prediction model for Individual Prognosis Or Diagnosis (TRIPOD+AI) guidance statement for prognostic studies using artificial intelligence.

Neonates were eligible for the HEAL Trial if they: (1) were at 36 weeks’ gestation or more at birth; (2) had 1 or more signs of perinatal depression (ie, Apgar score below 5 at 10 minutes; cardiorespiratory resuscitation beyond 10 minutes of age; pH level below 7.00 or with a base deficit of 15 mmol/L or more in a cord or infant gas within 60 minutes of age); (3) moderate or severe encephalopathy; and (4) hypothermia started within 6 hours of birth. Exclusion criteria for the HEAL Trial were birthweight below 1800 g, head circumference smaller than 30 cm, identified genetic or congenital condition affecting neurodevelopment, hematocrit above 65.0%, considering redirection of care, encephalopathy attributed to a postnatal event, and unlikely to be receive follow-up.^28^ Participants were additionally excluded from the current analysis a priori if they were later diagnosed with a congenital or genetic condition known to affect neurodevelopment before age 2 years, if they required extracorporeal membrane oxygenation (ECMO), or if neurophysiology results or outcome were not available.

### Clinical Predictors

Maternal and neonatal variables were collected from the medical record. Race and ethnicity (Hispanic or Latino, non-Hispanic) were determined by self-report; categories for race included American Indian or Alaskan Native, Asian, Black or African American, Native Hawaiian or other Pacific Islander, White, unknown, and not reported. Race and ethnicity were included because they have been shown to be a factor in birth outcomes. Severity of encephalopathy was classified as moderate or severe based on a modified Sarnat examination.^29^

### Electroencephalogram

For neonates with electroencephalogram (EEG) (97%) or amplitude-integrated EEG (aEEG) (3%) within 24 hours after birth, the clinical report was used to determine the presence of seizures and to classify the first described background at onset of recording as normal, excessively discontinuous, or severely abnormal using American Clinical Neurophysiology Society criteria.^30^ For 135 neonates (31%), EEG acquired throughout therapeutic hypothermia was reviewed by ^2^ pediatric neurophysiologists and the background scored at 5 time points as part of an ancillary study.^16^ The predominant background pattern was determined using the mean of the 5 EEG background ratings.^16^

### Magnetic Resonance Imaging

MRI was performed at 5 days of age (recommended imaging timing 96 to 144 hours after birth) using 3T MR scanners and standardized T1, T2, and diffusion-weighted imaging (DWI) sequences that were harmonized across sites.^31^ Two of 3 independent readers independently reviewed the images to determine the severity, location, and pattern of brain injury using a standardized scoring system,^32^ with discrepancies resolved by consensus.^31^

### Outcome

Examiners were trained and certified annually. Cognitive outcome was determined using the Bayley Scales of Infant Toddler Development, third edition (BSID-III) cognitive subscale. Motor outcome included cerebral palsy according to a standardized neurologic examination^33^ and the Gross Motor Function Classification System (GMFCS) score, modified for infants. GMFCS was assigned to describe the degree of functional disability and whether or not the infant had cerebral palsy (CP) by examination.^34^ Due to circumstances related to the COVID-19 pandemic, the assessment window was expanded, from the planned ages 22 to 26 months to ages 22 to 36 months.

The primary outcome was death or severe NDI (BSID-III cognitive score below 70, GMFCS 3 or higher, or quadriparesis with GMFCS 1 or higher) at age 2 years. A 5-level secondary outcome was defined as: no NDI, mild NDI, moderate NDI, severe NDI (as defined previously), or death, where severity was assigned using the worst of cognitive or motor outcome. BSID-III cognitive outcome was defined as: normal (≥90); mild (85-89); moderate (70-84). For motor outcome, hemiparesis or diparesis with GMFCS below 1 or no CP with GMFCS of 1 considered mild. Quadriparesis with GMFCS below 1, hemiparesis or diparesis with GMFCS 1 or 2, or no CP with GMFCS of 2 was considered moderate.

### Data Curation

From a study-wide dataset of 1404 variables, dates, study drug, adverse events, family history, placenta, outcome, and comments or descriptive variables were removed a priori (eFigure 1 in Supplement 1). We next removed 659 variables that were missing in 10% of participants or more, 481 variables with sparsity (ie, present in less than 10%), and 78 variables with low correlation (ρ < 0.1) with the primary outcome. When 2 or more variables were highly correlated, the most clinically relevant and widely accessible variable was retained. We derived 69 new variables to enhance clinical applicability.

### Statistical Analysis

Two recursive partitioning decision tree prediction models were developed (CTree method using the party library in R version 4.3.1 [R Project for Statistical Computing]) based on data available within the first day (for the 24 hour model) and after rewarming and MRI (postcooling model) (eTable 1 in Supplement 1).^35^ Data were split 70:30 into training and validation datasets with equal proportions of the primary outcome. Within the training dataset, 10-fold cross-validation was implemented to determine the simplest decision tree resulting in at least 1 group experiencing greater than 85% death or severe NDI (for both models). Infants not identified in the death or severe NDI group in the 24 hour model and who survived to the end of rewarming were included in the postcooling model. Performance metrics—sensitivity, specificity, positive and negative predictive values (PPV, NPV), and accuracy—with 95% CIs were assessed in both the training and validation datasets. CIs were derived using the Clopper-Pearson method for binomial distributions with the bdpv library in R using the actual prevalence of the outcome in each 2 × 2 table.^36^ CIs for multilevel outcomes were determined using the Wilson method in the binom library in R.

#### External Validation

For external validation, we analyzed a prospectively collected population-based UK cohort of 365 neonates at 36 weeks gestation or more fulfilling local guidelines for therapeutic hypothermia: (1) at least 1 of 10-minute Apgar score 5 or below, assisted ventilation 10 minutes after birth, pH level below 7.0, or base excess of −16 mmol/L or less in the first hour; (2) moderate or severe encephalopathy; or (3) moderately or severely abnormal aEEG or 3 or more minutes of aEEG seizures per hour within 6 hours of birth. Model variables were similar between cohorts except aEEG, which was only available for less than 6 hours after birth. Burst suppression, low amplitude, and flat trace were defined as severely abnormal. MRI basal ganglia or thalamus injury was scored according to Rutherford et al^37^ using T1, T2, and DWI when available, which was similar to the HEAL methods.^38^ BSID-III motor or cognitive subscale below 70 or GMFCS above 2 at ages 18 to 24 months was considered severe NDI.

Missing clinical data were imputed with the median value. Missing MRI values for infants who survived to day 5 (8 training, 8 internal validation, and 11 external validation) were imputed with the highest (worst) scores, assuming clinical instability as the reason for missing imaging data.

#### Sensitivity Analyses

We performed sensitivity analyses to examine the predictive accuracy of the final decision tree based on the following: (1) timing of MRI (within or after 7 days after birth), (2) ignoring the 24-hour model (ie, including all neonates who survived to the end of cooling), (3) using only the available MRI data without imputation, and (4) combining the HEAL and UK datasets for the postcooling model only on infants who survived to discharge.

## Results

Among 500 neonates enrolled in the HEAL trial, 424 (84.8%) were included in the predictive modeling dataset (mean [SD] gestational age, 39.1 [1.4] weeks; 192 female [45.3%]; 28 Asian [6.6%], 50 Black [11.8%], 311 White [73.3%]); 105 infants (24.7%) had severe encephalopathy at enrollment. A priori exclusions were as follows: loss to follow-up (38 infants [7.6%]), ECMO (22 infants [4.4%]), diagnosis of a congenital or genetic condition known to affect neurodevelopment (21 infants [4.2%]), and no EEG or aEEG available (13 infants [2.6%]). The primary outcome of death or severe NDI was present in 105 infants (24.8%): 59 (13.9%) died and 46 (10.8%) had severe NDI. Multiple clinical, EEG, and imaging factors were associated with death or severe NDI (Table 1).

**Table 1.** Clinical, EEG, and MRI Characteristics of 424 Neonates With Hypoxic-Ischemic Encephalopathy With and Without Death or Severe NDI at Age 2 Years

Characteristics	No. (%)			*P* value^a^
	Total (N = 424)	NDI		
		Death or severe (n = 105)	None, mild, or moderate (n = 319)	
**Delivery and resuscitation**				
Sentinel event^b^	129 (30.4)	38 (36.1)	91 (28.5)	.14
Urgent or emergent cesarean section delivery	268 (63.2)	75 (71.4)	193 (60.5)	.04
Epinephrine or resuscitation >10 min	385 (90.8)	102 (97.1)	283 (89.7)	.01
**Infant clinical characteristics**				
Sex				
Female	192 (45.3)	49 (46.7)	143 (44.8)	.74
Male	232 (54.7)	56 (53.3)	176 (55.2)	
Hispanic ethnicity	100 (23.6)	22 (20.9)	78 (24.4)	.46
Race				
Asian	28 (6.6)	3 (2.8)	25 (7.9)	.17
Black or African American	50 (11.8)	14 (13.3)	36 (11.3)	
White	311 (73.3)	76 (72.3)	235 (73.7)	
Other or multiple^c^	35 (8.2)	12 (11.4)	23 (7.3)	
Birth weight, mean (SD), g	3374 (591)	3369 (605)	3376 (588)	.92
Gestational age, mean (SD), wk	39.1 (1.4)	39.0 (1.5)	39.2 (1.4)	.26
5-min Apgar score, median (IQR)	3 (2-5)	2 (0-3)	4 (2-5)	<.001
Lowest pH, mean (SD)^d^	6.9 (0.2)	6.8 (0.2)	7.0 (0.2)	<.001
Worst base deficit, mean (SD), mmol/L^d^	18.3 (5.6)	21.5 (7.2)	17.4 (5.6)	<.001
Severe encephalopathy at enrollment^e^	105 (24.8)	61 (58.1)	44 (13.8)	<.001
No. of severe Sarnat exam categories at enrollment, median (IQR)	1 (1-3)	3 (2-5)	1 (0-2)	<.001
Maximum glucose first 24 h mean (SD), mg/dL	174.5 (74.4)	219.9 (88.9)	159.6 (62.4)	<.001
Minimum glucose first 24 h mean (SD), mg/dL	91.1 (40.1)	106.3 (49.9)	85.7 (34.5)	<.001
Intubated or ventilated	339 (80.0)	100 (95.2)	239 (74.9)	<.001
ALT ≥100, U/L	178 (42.0)	73 (69.5)	105 (32.9)	<.001
Creatinine >1.5 mg/DL	41 (9.7)	26 (24.8)	15 (4.7)	<.001
Clinical EEG report				
EEG background, first 24 h^f^				
Normal	172 (40.6)	7 (6.7)	165 (51.7)	<.001
Discontinuous	141 (33.3)	19 (18.1)	122 (38.2)	
Severely abnormal	111 (26.2)	79 (75.2)	32 (10.0)	
Seizures within first 24 h	96 (22.6)	48 (45.7)	48 (15.0)	<.001
MRI				
Patients, No.	402	86	316	
MRI day of life, median (IQR)	4.9 (4.5-5.6)	4.8 (4.2-5.4)	5.0 (4.5-5.6)	.19
Thalamus T1, T2, DWI abnormality	170 (42.3)	79 (91.9)	91 (28.8)	<.001
Caudate T1, T2, DWI abnormality	71 (17.7)	56 (65.1)	15 (4.7)	<.001
Putamen/globus pallidus T1, T2, DWI abnormality	181 (45.0)	77 (89.5)	104 (32.6)	<.001
Post-MRI Sarnat examination				
Patients, No.	401	90	311	
No. of severe Sarnat examination category results, median (IQR)	0 (0-1)	2 (1-5)	0 (0-0)	<.001

Abbreviations: ALT, alanine transferase; DWI, diffusion weighted imaging; EEG, electroencephalogram; MRI, magnetic resonance imaging; NDI, neurodevelopmental impairment.

SI conversion factor: To convert ALT to microkats per liter, multiply by 0.0167; creatinine to micromoles per liter, multiply by 88.4; glucose to millimoles per liter, multiply by 0.0555.

^a^ Unadjusted *P* values were calculated using 2-sample *t* test for continuous variables and χ^2^ tests for categorical variables, or Fisher exact tests when tabulated counts were 5 or less.

^b^ Sentinel event indicates placental abruption, shoulder dystocia, uterine rupture, or prolapsed cord.

^c^ Other included American Indian or Alaska Native, unknown, and not reported.

^d^ Lowest pH and worst base deficit among cord arterial, cord venous, and arterial blood gas samples taken before 60 minutes of age.

^e^ Severe encephalopathy as defined by modified Sarnat score.

^f^ Severely abnormal EEG defined as burst suppression, low voltage suppressed, or status epilepticus.

### Twenty-Four–Hour Model

**Figure 1.**
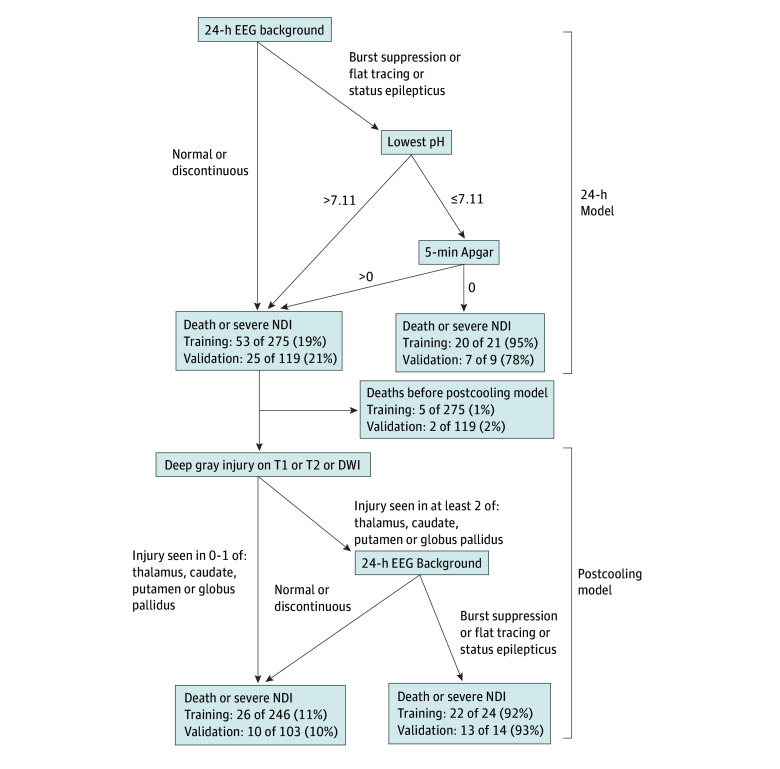
Models Predicting Death or Severe Neurodevelopmental Impairment (NDI) at Age 2 Years With Hypoxic-Ischemic Encephalopathy DWI indicates diffusion-weighted imaging; EEG, electroencephalogram.

We first created a recursive partitioning model using the curated set of 12 variables that were available at 24 hours of age. Three factors were highly predictive of death or severe NDI: (1) severely abnormal EEG (burst suppression, flat tracing, or status epilepticus) within the first 24 hours, (2) lowest cord or 60-minute pH level of 7.11 or lower, and (3) 5-minute Apgar equal to 0 (Figure 1). The presence of all 3 of these factors had a specificity of 99.6% (95% CI, 97.5%-100%) and a PPV 95.2% (95% CI, 73.2%-99.3%) for death or severe NDI in the training dataset, and a specificity of 97.9% (95% CI, 92.7%-99.8%) with a PPV 77.8% (95% CI, 43.4%-94.1%) in the internal validation dataset (Table 2).

**Table 2.** Training and Validation Test Metrics for 24-Hour and Postcooling Models for Death or Severe Neurodevelopmental Impairment at Age 2 Years

Dataset or metric	24-h model, % (95% CI) (N = 424)	Postcooling model, % (95% CI) (n = 387)
**Training**		
Sensitivity	27.4 (17.6-39.1)	45.8 (31.4-60.8)
Specificity	99.6 (97.5-100)	99.1 (96.8-99.9)
Positive predictive value	95.2 (73.2-99.3)	91.7 (72.8-97.8)
Negative predictive value	80.6 (78.3-82.7)	89.4 (86.7-91.7)
Accuracy	81.7 (76.8-85.9)	89.6 (85.4-93.0)
**Internal validation**		
Sensitivity	21.9 (9.3-40.0)	56.5 (34.5-76.8)
Specificity	97.9 (92.7-99.8)	98.9 (94.2-100)
Positive predictive value	77.8 (43.4-94.1)	92.9 (64.2-99.0)
Negative predictive value	79.0 (75.8-81.9)	90.3 (85.4-93.7)
Accuracy	78.9 (70.8-85.6)	90.6 (83.8-95.2)
**External validation** ^a^		
Sensitivity	6.6 (2.5-13.8)	38.5 (25.3-53.0)
Specificity	97.6 (95.1-99.0)	98.6 (96.5-99.6)
Positive predictive value	46.2 (23.3-70.8)	83.3 (64.1-93.4)
Negative predictive value	77.1 (76.1-78.1)	89.8 (87.7-91.6)
Accuracy	76.0 (71.5-80.2)	89.4 (85.6-92.5)

^a^ The external validation cohort was a prospectively collected UK cohort of 365 neonates at ≥36 weeks gestation.

### Postcooling Model

We then created a recursive partitioning model using a curated set of 40 variables that were available after therapeutic hypothermia and MRI (Figure 1). Seven children who were not predicted to have death or severe NDI in the 24-hour model died prior to rewarming and MRI and, therefore, were excluded from the postcooling model (5 of 275 [0.7%] in the training dataset and 2 of 119 [1.7%] in the validation dataset).

In the postcooling model, T1, T2, or diffusion-weighted imaging (DWI) abnormality on MRI in at least 2 of 3 deep gray regions (ie, thalamus, caudate, and putamen or globus pallidus) combined with a severely abnormal EEG in the first 24 hours had a specificity of 99.1% (95% CI, 96.8%-99.9%) and PPV of 91.7% (95% CI, 72.8%-97.8%) for death or severe NDI within the training dataset, and a specificity of 98.9% (95% CI, 94.1%-100%) and PPV of 92.9% (95% CI, 64.2%-99.0%) in the internal validation dataset (Table 2).

**Figure 2.**
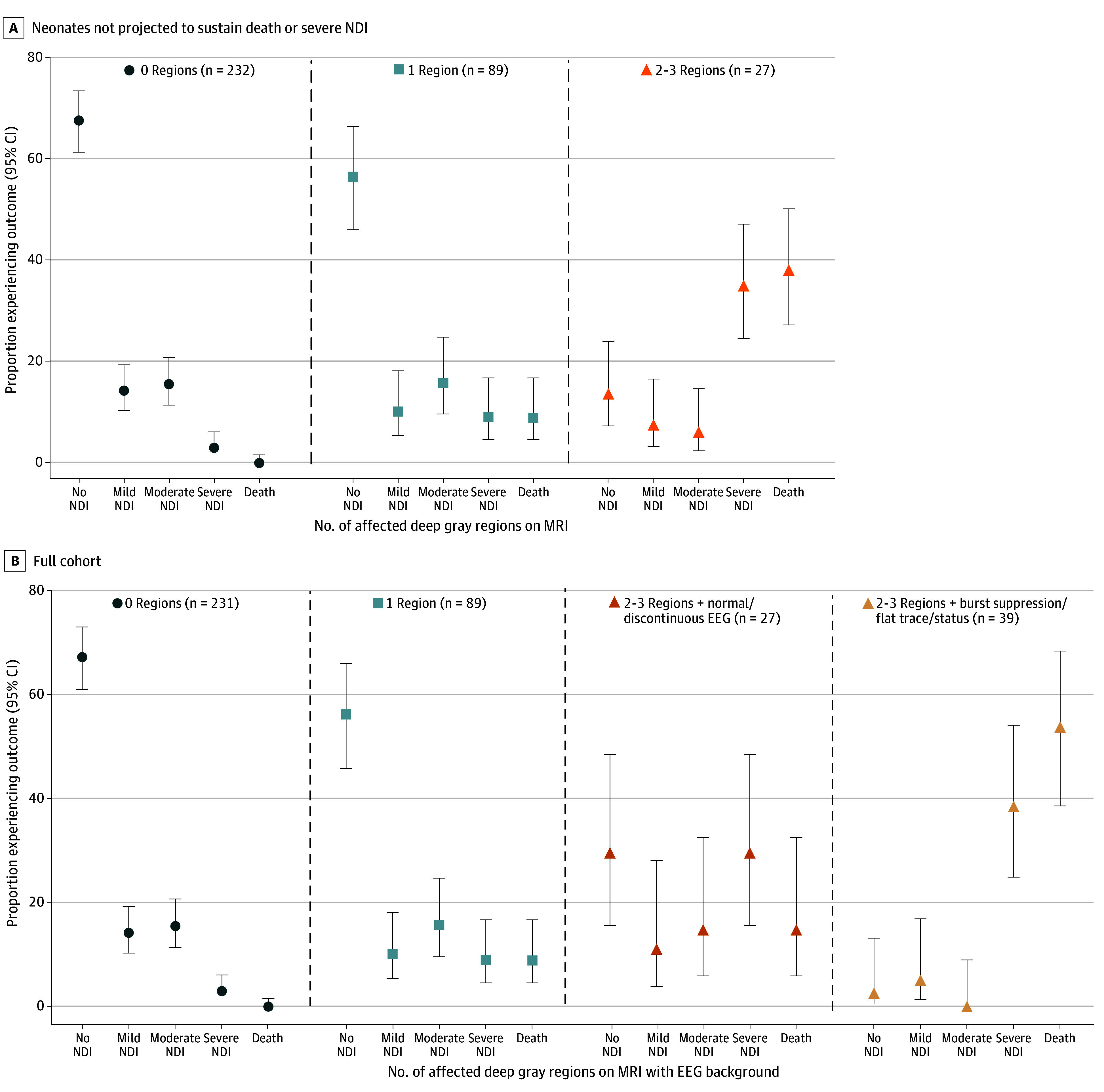
Neurodevelopmental Outcome Among the Neonates Not Predicted to Sustain Death or Severe Neurodevelopmental Impairment (NDI) and for the Full Cohort In panel A, a total of 349 neonates who were alive after cooling and magnetic resonance imaging and not predicted to sustain death or severe NDI by number of deep gray regions (thalamus, caudate, globus pallidus/putamen) with injury on T1, T2, or diffusion-weighted imaging; B, includes the full cohort and adds 24-hour electroencephalogram (EEG) background results.

**Figure 3.**
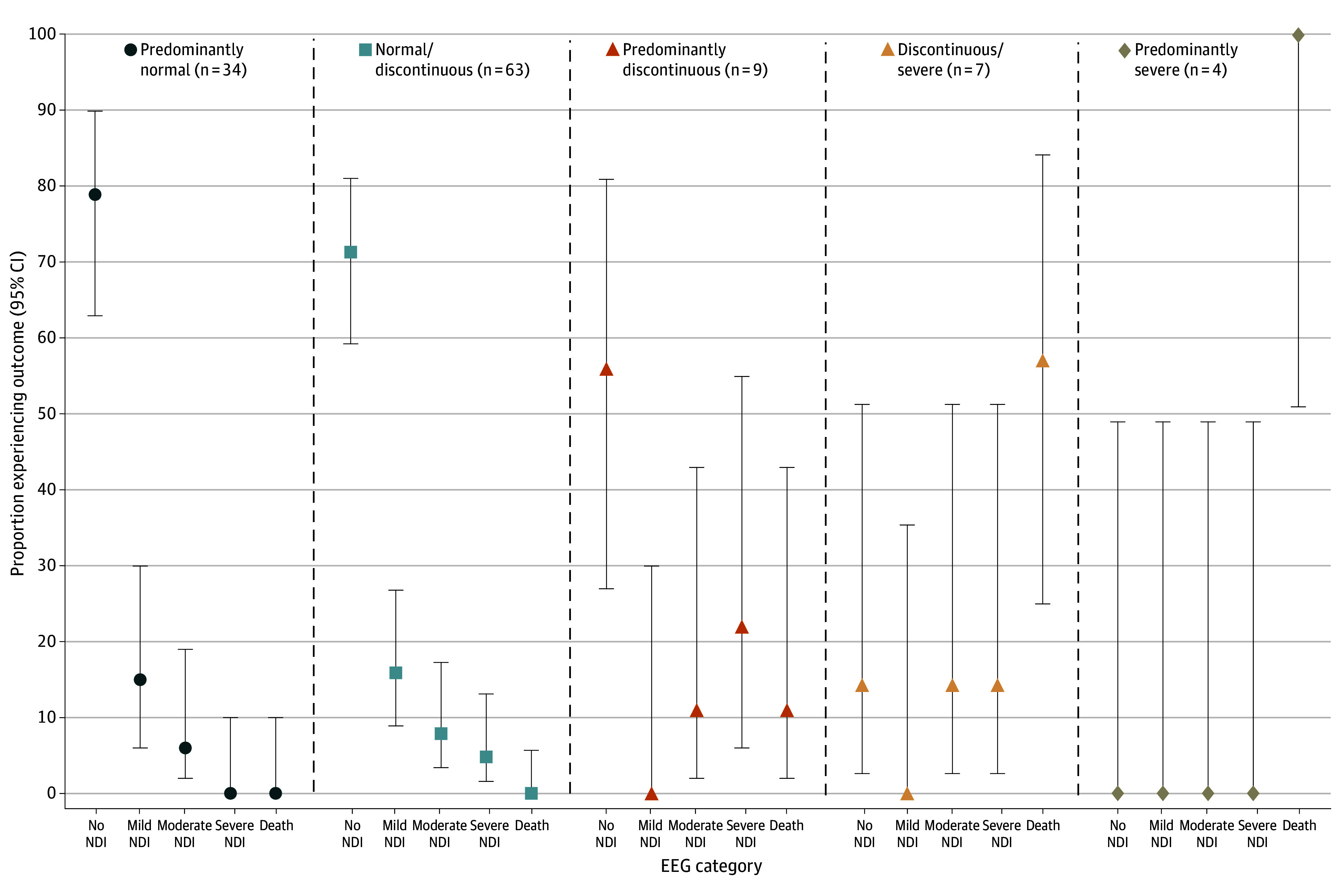
Neurodevelopment Among Neonates Not Predicted to Sustain Death or Severe Neurodevelopmental Disability (NDI) by Electroencephalography (EEG) Throughout Cooling A total of 117 neonates are included in the cohort not predicted to sustain death or severe NDI.

Individual outcomes of the surviving infants who were predicted to have death or severe NDI are presented in eTable 2 in Supplement 1. Stratified analyses for the 5-level outcome by MRI and 24-hour EEG background (Figure 2) and by predominant EEG background throughout cooling (Figure 3) provide additional information about likelihood of each outcome. Review of the MRI results among children predicted to but who did not have death or severe NDI (ie, false-positive MRI) demonstrated either very small areas of injury that spanned multiple scored regions (2 infants; eFigure 2 in Supplement 1) or had abnormal signal that could be interpreted as pre-Wallerian or secondary degeneration (eg, reduced diffusion in the dorsal thalami instead of the ventrolateral thalami (1 infant).

### Sensitivity Analyses

Three sensitivity analyses of the postcooling model used the training and validation datasets combined: MRI before vs after day 7 (144 hours) among neonates who were not predicted to experience death or severe NDI by the 24-hour model, infants who survived to the end of cooling (ie, including those with predicted death or severe NDI in the 24-hour model, using complete MRI data only without imputation). An additional sensitivity analysis included only the infants who survived to hospital discharge from the HEAL and UK datasets. Specificity and positive predictive value were between 88% and 100% in the sensitivity analyses (eTable 3 in Supplement 1).

### External Validation

In the external validation dataset, 34 or 365 died (9.3%) and 37 had severe NDI (10.1%). Specificity was above 97.6% in both the 24-hour and postcooling models, whereas PPV was 46.2% in the 24-hour model and 83.3% in the postcooling model (Table 2).

## Discussion

Using data from the HEAL trial, we found that several clinically available findings, when used in combination, provided excellent specificity and positive predictive value for death or severe NDI at age 2 years in infants with perinatal HIE treated with hypothermia. Our models improve upon prior studies by using an agnostic recursive partitioning approach in a large, multicenter dataset to develop a comprehensive predictive model that combines clinical, neurophysiology, and neuroimaging factors at 2 distinct and clinically relevant time points. The models retained excellent model metrics in an independent dataset, particularly for the postcooling model, suggesting strong external validity.

Although numerous prior studies have identified factors that are associated with adverse neurodevelopmental outcomes in neonates with HIE,^2,3,4,5,6,7,8,9,10,11,12,13,14,15,16,17,18,19,20,21,22,23,24,25,26^ few have attempted to combine clinical, neurophysiology, and imaging factors to develop a validated, comprehensive, and simple to interpret predictive model that can be used at the bedside. Our recursive partitioning model identified that a severely abnormal EEG along with low pH and 5-minute Apgar of 0 (variables that are widely available in the first 24 hours after birth) and MRI injury to at least 2 deep gray regions (findings that are typically available within 4-5 days of birth), had excellent specificity and PPV for death or severe NDI.

The association between EEG metrics and neurodevelopmental outcome in neonates with HIE has been recognized for decades.^39,40,41,42^ In a 2023 study,^16^ we showed that severity and duration of EEG background abnormalities are associated with neurodevelopmental outcome. Multiple prior studies have shown that MRI is associated with adverse outcomes in infancy and childhood.^17,37,38,43,44,45,46^ The automated model selected injury to the deep gray nuclei but not white matter or cortical injury. A watershed pattern of injury typically manifests clinically later in development as impaired language and cognition,^47,48^ and so injury to the watershed areas including white matter and cortex may be more relevant to prediction models for school age or adolescent outcomes.

The models were not designed to predict children with no or mild NDI. However, we present outcomes stratified by MRI and EEG severity, which show that between 75% and 95% had normal neurodevelopment or only mild NDI at age 2 years if MRI or EEG were normal or only mildly abnormal (ie, without injury to or with only 1 area of affected area of the deep gray nuclei; normal or discontinuous EEG). This information may be reassuring to families.

Death in the intensive care unit (ICU) can involve a decision not to initiate or to withdraw life-sustaining treatment in the context of anticipated poor neurologic prognosis.^49,50,51^ An inherent limitation of any model predicting death is the possibility of a self-fulfilling prophecy, as life-sustaining treatment may be withdrawn for patients with severe prognostic indicators.^52^ Although we speculate that neonates in this cohort with severely abnormal EEG or MRI injury who died following withdrawal of life-sustaining treatment would have sustained severe NDI if they were maintained on life-sustaining treatments, we cannot be certain. Also, the composite outcome of death or severe NDI weights death and severe NDI equally, despite each component being valued differently by many clinicians and parents.^53,54,55^

### Limitations

Although we present models with high internal and external validation, our results are not without limitations. First, we developed models to optimize specificity rather than selecting models that balance sensitivity and specificity. The decision was made to allow the bedside clinician to provide a likelihood of death or disability with a high degree of certainty, although this approach limits the ability to counsel families about the anticipated outcome among children without the risk factors identified in the models, particularly at 24 hours where sensitivity was low. For these children, we provide guidance regarding the likelihood and severity of NDI based on EEG and MRI results (Figures 2 and 3). Second, our data were bound by exclusion criteria for the parent HEAL Trial (particularly anticipated transition to palliative care). The high specificity and PPV of the postcooling model in the external validation dataset suggests this limitation had a negligible impact on model validity. Third, the external validation data element definition for aEEG was different from the HEAL Trial (external validation dataset used only aEEG within the first 6 hours after birth rather than the first 24 hours), which may account for lower specificity since aEEG recovery during this time is associated with a favorable prognosis among neonates who undergo hypothermia.^56,57^ Fourth, sensitivity analyses combined the training and validation datasets. However, as these analyses pertain to factors that might affect MRI in the postcooling model and performance of the postcooling model was very similar in the training and validation datasets, combining them is unlikely to have resulted in an artificial improvement in performance in the sensitivity analyses. Fifth, only the results from the initial 24 hours of EEG were available for the recursive partitioning models. While some studies have shown that 48-hour EEG is most predictive for neurodevelopment,^58^ we recently showed that 24-hour EEG is also highly associated with death and severe disability.^16^ Multiday EEG results may be important to further refine the models for death or severe NDI and to develop predictive models for mild or moderate disability. Sixth, MR spectroscopy (MRS) may improve model metrics.^18^ However, since MRS is not routinely acquired, we did not include it in the model. Also, the MRI scoring system did not assess the exact location within deep gray nuclei, nor differentiate between whether signal abnormality is thought to be due to primary injury or secondary degeneration. This led to a small number of false positive cases. Expert neuroradiology interpretation remains important. Finally, what is considered severe NDI may differ based on clinician and family experience, culture, and values. For this study, severe NDI was defined a priori according to definitions developed for the HEAL trial.^28^

The predictive models in this paper should not be used as definitive forecast of an individual child’s developmental outcome. Rather, they are 1 tool that can facilitate prognostic communication within the health care team and with families. Prognostic models should be used within a clear communication strategy, for example, the ALIGN framework and our-HOPE, which were developed for use with families of infants with neurologic conditions.^59,60^ Communication with families must also acknowledge that developmental prediction using data collected during the neonatal admission is inherently uncertain. Increasing evidence points to the importance of the home environment for neurodevelopment; intensive, targeted, and goal-directed therapies can impact developmental trajectory in infants at risk for disability.^61^ Furthermore, socioeconomic status, parental language preference, parent well-being, and parental education are associated with brain growth and development,^62^ and neurodevelopmental outcome^63,64^ in diverse high-risk populations. Altogether, these data suggest that neurodevelopmental outcome is, to at least some degree, modifiable, and every effort must be made to enable a developmentally supportive environment.

## Conclusions

In this prognostic study, we developed a predictive model with high specificity and PPV for death or severe NDI using simple, readily available clinical, EEG, and MRI results in 2 independent datasets. These models may be used to counsel families of newborns with HIE about anticipated death or severe NDI within days after birth.

## Notes

Supplement 1. **eTable 1.** Variables Considered for Inclusion in the 24h and Postcooling Models **eTable 2.** Outcome at Age 2 Years for All Surviving Infants Who Were Predicted to Have Death or Severe NDI for the 24h Model and the Postcooling Model **eTable 3.** Sensitivity Analyses to Evaluate Test Metrics for the Postcooling Model Based on Timing of MRI (Within or After 7 Days After Birth), Ignoring the 24 Hours Model (ie, Including All Neonates Who Survived to the End of Cooling), Using Only the Available MRI Data Without Imputation, and Including Only the Infants That Survived to Hospital Discharge **eFigure 1.** HEAL Trial Candidate Prediction Variable Selection Flow Diagram **eFigure 2.** Imaging From a False Positive MRI Results

Supplement 2.
Data Sharing Statement
