# Errors in Figures

**DOI:** 10.1001/jamanetworkopen.2024.59716

**Published:** 2025-01-09

In the Original Investigation titled “Predictors of Death or Severe Impairment in Neonates With Hypoxic-Ischemic Encephalopathy,”^1^ published December 5, 2024, typos were included in labels of Figure 1. These have been fixed, and a detail clarifying the sample in panel B of Figure 2 has been added. This article has been corrected.^1^

## References

[1] Glass HC, Wood TR, Comstock BA, . Predictors of death or severe impairment in neonates with hypoxic-ischemic encephalopathy. JAMA Netw Open. 2024;7(12):e2449188. doi:10.1001/jamanetworkopen.2024.49188 39636636 PMC11621987

