# Error in Table 1

**DOI:** 10.1001/jamanetworkopen.2025.57296

**Published:** 2026-01-09

In the Original Investigation titled “Predictors of Death or Severe Impairment in Neonates With Hypoxic-Ischemic Encephalopathy,”^1^ published December 5, 2024, data for magnetic resonance imaging results were transposed between columns 3 and 4 of Table 1. This article has been corrected.^1^
