# Supplementary material for: Predictors of Death or Severe Impairment in Neonates With Hypoxic-Ischemic Encephalopathy
Source: JAMA Netw Open. 2024 Dec 5;7(12):e2449188. doi: 10.1001/jamanetworkopen.2024.49188 (PMC11621987; doi:10.1001/jamanetworkopen.2024.49188)

Supplemental Online Content

Glass HC, Wood TR, Comstock BA, et al. Predictors of death or severe impairment in neonates with hypoxic-ischemic encephalopathy. *JAMA Netw Open.* 2024;7(12):e2449188. doi:10.1001/jamanetworkopen.2024.49188

eTable 1. Variables Considered for Inclusion in the 24h and Postcooling Models

eTable 2. Outcome at Age 2 Years for All Surviving Infants Who Were Predicted to Have Death or Severe NDI for the 24h Model and the Postcooling Model

eTable 3. Sensitivity Analyses to Evaluate Test Metrics for the Postcooling Model Based on Timing of MRI (Within or After 7 Days After Birth), Ignoring the 24 Hours Model (ie, Including All Neonates Who Survived to the End of Cooling), Using Only the Available MRI Data Without Imputation, and Including Only the Infants That Survived to Hospital Discharge

eFigure 1. HEAL Trial Candidate Prediction Variable Selection Flow Diagram

eFigure 2. Imaging From a False Positive MRI Results

This supplemental material has been provided by the authors to give readers additional information about their work.

© 2024 Glass HC et al. *JAMA Network Open.*

Tables

eTable 1

Variables considered for inclusion in the 24h and Post-Cooling models.

Variable Type	Variable	Correlation with Outcome	Model (Opportunity for Selection)
Clinical	Urgent or emergent delivery by Cesarean section	0.12	24h + Post-Cooling
Clinical	5-minute Apgar score	-0.27	24h + Post-Cooling
Clinical	Baseline Sarnat - number of severe items (out of 6: LOA, LOC, posture, muscle tone, moro or suck, pupils or respiration)	0.48	24h
Clinical	Baseline Sarnat Respiratory Status	0.28	24h + Post-Cooling
Clinical	Day 5 Sarnat - number of moderate/severe neuro categories (activity, consciousness, tone, posture)	0.63	Post-Cooling
Clinical	Day 5 Sarnat - number of severe items (out of 6: LOA, LOC, posture, muscle tone, moro or suck, pupils or respiration)	0.66	Post-Cooling
Clinical	Day 5 Sarnat - number of severe neuro categories (activity, consciousness, tone, posture)	0.58	Post-Cooling
Clinical	Gestational age at birth	-0.12	Post-cooling
Clinical	Intubated at time of Sarnat exam or rp_respfail==1	0.21	24h + Post-Cooling
Clinical	Lowest pH cord or 1st 24h	-0.27	24h + Post-Cooling
Clinical	Maximum glucose in 1st 24h	0.32	24h
Clinical	Maximum glucose in 1st 72h	0.33	Post-Cooling
Clinical	Minimum glucose in 1st 24h	0.19	24h
Clinical	Minimum glucose in 1st 72h	0.10	Post-Cooling
Clinical	Resucitation >10min or epinephrine	0.12	Post-Cooling
Clinical	Sentinel event (shoulder dystocia, prolapse cord, uterine rupture, or placental abruption)	0.13	24h + Post-Cooling
Clinical	Worst base deficit cord or 1st 24h	-0.25	24h + Post-Cooling
EEG	EEG background, 1st 24 hours by report	0.59	24h + Post-Cooling
EEG	EEG seizures 1st 24h by report	0.28	24h + Post-Cooling
MRI	Putamen/globus pallidus, Thalamus, Caudate abnormality (DWI, T1, or T2), number of regions, 0-3	0.57	Post-Cooling
MRI	Putamen/globus pallidus, Thalamus, Caudate, Brainstem abnormality (DWI, T1, or T2), number of regions, 0-4	0.63	Post-Cooling
MRI	Putamen/globus pallidus, Thalamus, Caudate abnormality (DWI only), number of regions, 0-3	0.69	Post-Cooling
MRI	Putamen/globus pallidus, Thalamus, Caudate, Brainstem abnormality (DWI only), number of regions, 0-4	0.70	Post-Cooling
MRI	Caudate abnormality (DWI, T1, or T2), any	0.60	Post-Cooling
MRI	Caudate abnormality (DWI), any	0.61	Post-Cooling
MRI	Cortex abnormality (DWI), numeric	0.60	Post-Cooling
MRI	Cortex abnormality (DWI, T1, or T2), severe	0.48	Post-Cooling
MRI	Cortex abnormality (DWI, T1, or T2), any	0.48	Post-Cooling
MRI	Cortex abnormality (DWI, T1, or T2), moderate/severe	0.57	Post-Cooling
MRI	Cortex abnormality (DWI), severe	0.48	Post-Cooling
MRI	Cortex abnormality (DWI), any	0.50	Post-Cooling
MRI	Cortex abnormality (DWI), moderate/severe	0.58	Post-Cooling
MRI	Normal MRI	-0.31	Post-Cooling
MRI	Putamen/globus pallidus abnormality (DWI, T1, or T2)	0.42	Post-Cooling
MRI	Putamen/globus pallidus abnormality (DWI)	0.62	Post-Cooling
MRI	Thalamus abnormality (DWI, T1, or T2), any	0.61	Post-Cooling

MRI	Thalamus abnormality (DWI), any	0.49	Post-Cooling
MRI	White matter abnormality (DWI, T1 or T2), any	0.32	Post-Cooling
MRI	White matter abnormality (DWI, T1 or T2), moderate/severe	0.52	Post-Cooling
MRI	White matter abnormality (DWI, T1 or T2), severe	0.57	Post-Cooling
MRI	White matter abnormality (DWI), any	0.36	Post-Cooling
MRI	White matter abnormality (DWI), moderate/severe	0.50	Post-Cooling
MRI	White matter abnormality (DWI), severe	0.53	Post-Cooling

© 2024 Glass HC et al. *JAMA Network Open.*

eTable 2

Outcome at age two years for all surviving infants who were predicted to have death or severe NDI for the 24h Model (**A**) and the Post-Cooling Model (**B**). The models also correctly predicted death of 20 (17 training, 3 validation) neonates for the 24h Model and 25 (16 training, 9 validation) for the Post-Cooling Model (not shown).

A)

Outcome	Bayley Cognitive	Cerebral Palsy (CP) Status	GMFCS
True Positive			
Severe NDI	55	Quadriparesis	3
Severe NDI	55	Quadriparesis	5
Severe NDI	55	Quadriparesis	5
Severe NDI	54	Quadriparesis	5
False Positives			
Moderate NDI	70	No CP	0
Mild NDI	85	No CP	0
Normal	95	No CP	0.5

B)

Outcome	Bayley Cognitive	Cerebral Palsy (CP) Status	GMFCS
True Positive			
Severe NDI	55	Quadriparesis	2
Severe NDI	55	Diparesis	1
Severe NDI	55	No CP	0
Severe NDI	65	No CP	0
Severe NDI	60	No CP	0
Severe NDI	55	Quadriparesis	5
Severe NDI	85	Quadriparesis	3
Severe NDI	55	No CP	0
Severe NDI	55	Quadriparesis	4
Severe NDI	55	Quadriparesis	4
False Positive			
Normal	85	No CP	0
Mild NDI	85	No CP	0
Mild NDI	90	No CP	0

NDI neurodevelopmental impairment; **CP** cerebral palsy, **GMFCS** gross motor function classification system

eTable 3

Sensitivity analyses to evaluate test metrics for the Post-Cooling Model **A)** based on timing of MRI (within or after seven days after birth), **B)** ignoring the 24 hours model (*i.e.,* including all neonates who survived to the end of cooling), **C)** using only the available MRI data without imputation, and **D)** including only the infants that survived to hospital discharge.

A)	**Metric**	**Post-Cooling Model**
**MRI <7 days predicted to have death or severe NDI (n=29/323)**	Sensitivity	50.0% (36.1-63.9%)
	Specificity	99.3% (97.3-99.9%)
	Positive Predictive Value	93.1% (76.8-98.2%)
	Negative Predictive Value	90.8% (88.3-92.8%)
	Accuracy	91.0% (87.4-93.9%)
**MRI ≥7 days predicted to have death or severe NDI (n=5/55)**	Sensitivity	40.0% (12.2-73.8%)
	Specificity	97.8% (88.2-99.9%)
	Positive Predictive Value	80.0% (33.3-97.0%)
	Negative Predictive Value	88.0% (81.5-92.4%)
	Accuracy	87.3% (75.5-94.7%)

**B)**	**Metric**	**Post-Cooling Model**
**Survived to the end of cooling (n=49/413)**	Sensitivity	48.9% (38.5-59.5%)
	Specificity	99.1% (97.3-99.8%)
	Positive Predictive Value	93.9% (83.0-98.0%)
	Negative Predictive Value	86.8% (84.4-88.9%)
	Accuracy	87.7% (84.1-90.7%)

**C)**	**Metric**	**Post-Cooling Model**
**Using only the available MRI data without imputation (n=34/377)**	Sensitivity	48.4% (35.8-61.3%)
	Specificity	99.0% (97.2-99.8%)
	Positive Predictive Value	91.2% (76.5-97.0%)
	Negative Predictive Value	90.4% (88.1-92.3%)
	Accuracy	90.5% (87.0-93.2%)

© 2024 Glass HC et al. *JAMA Network Open.*

D)	Metric	Post-Cooling Model
**Combining the HEAL and UK data sets for infants who survived to discharge (n=94/707)**	Sensitivity	42.6% (32.4-53.2%)
	Specificity	98.7% (97.4-99.4%)
	Positive Predictive Value	83.3% (70.7-91.2%)
	Negative Predictive Value	91.8% (90.4-93.0%)
	Accuracy	91.8% (88.9-93.2%)

Figures

eFigure 1

HEAL Trial candidate prediction variable selection flow diagram.

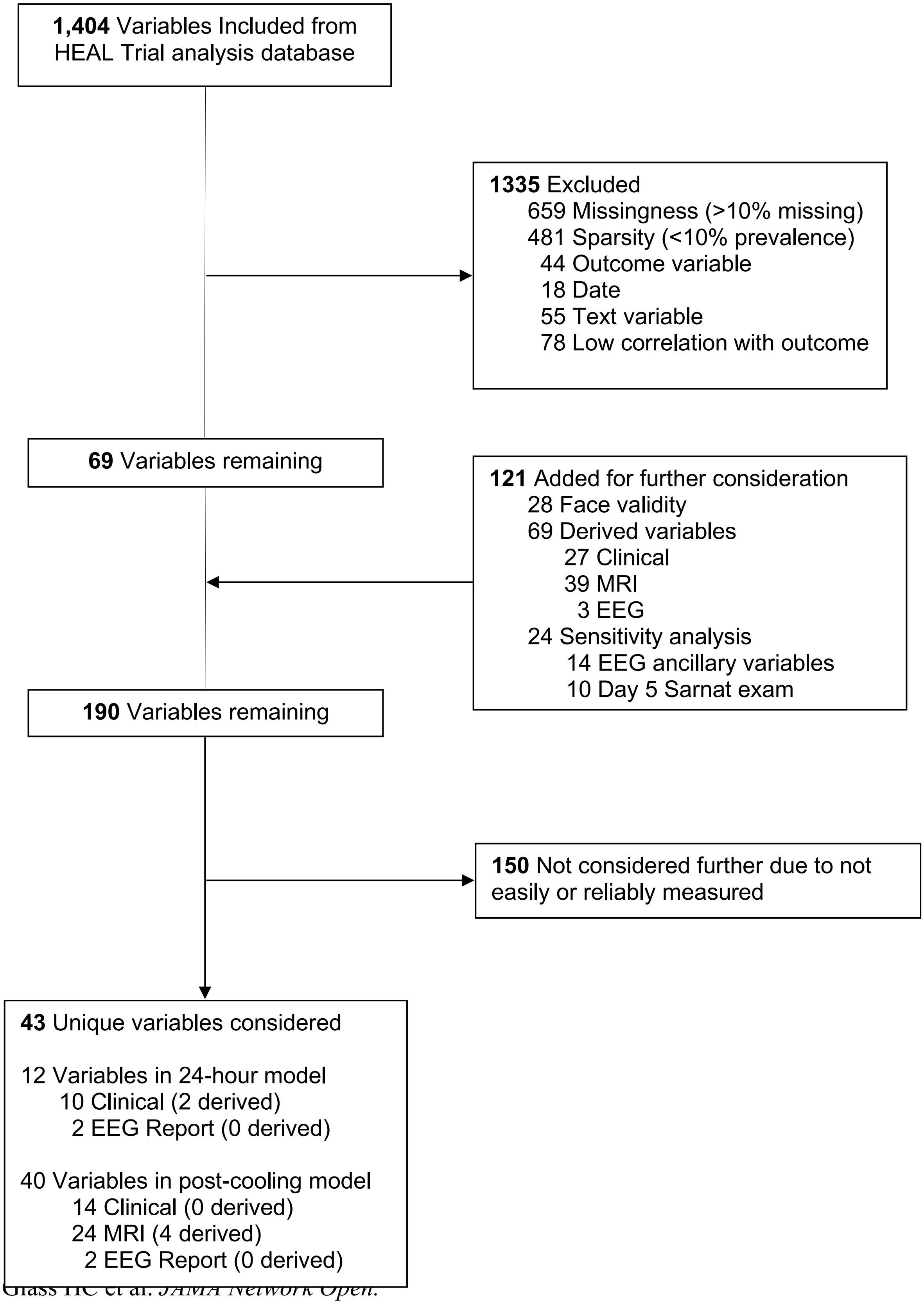

© 2024 Glass HC et al. *JAMA Network Open.*

eFigure 2

Imaging from a false positive MRI results. **A)** Axial ADC map demonstrating small focal regions of reduced diffusion in the right caudate head (long white arrow) and right globus pallidus (short white arrow), constituting two regions of involvement. **B)** Axial ADC map demonstrating reduced diffusion involving the right putamen (white arrow), and bilateral thalami (white arrowheads). The abnormality involves the dorsal thalami rather than the ventrolateral thalami (which would be a hallmark of acute profound hypoxic-ischemic injury in a term newborn), suggesting pre-Wallerian or secondary degeneration rather than primary injury. These regions of reduced diffusion constitute two regions of involvement.

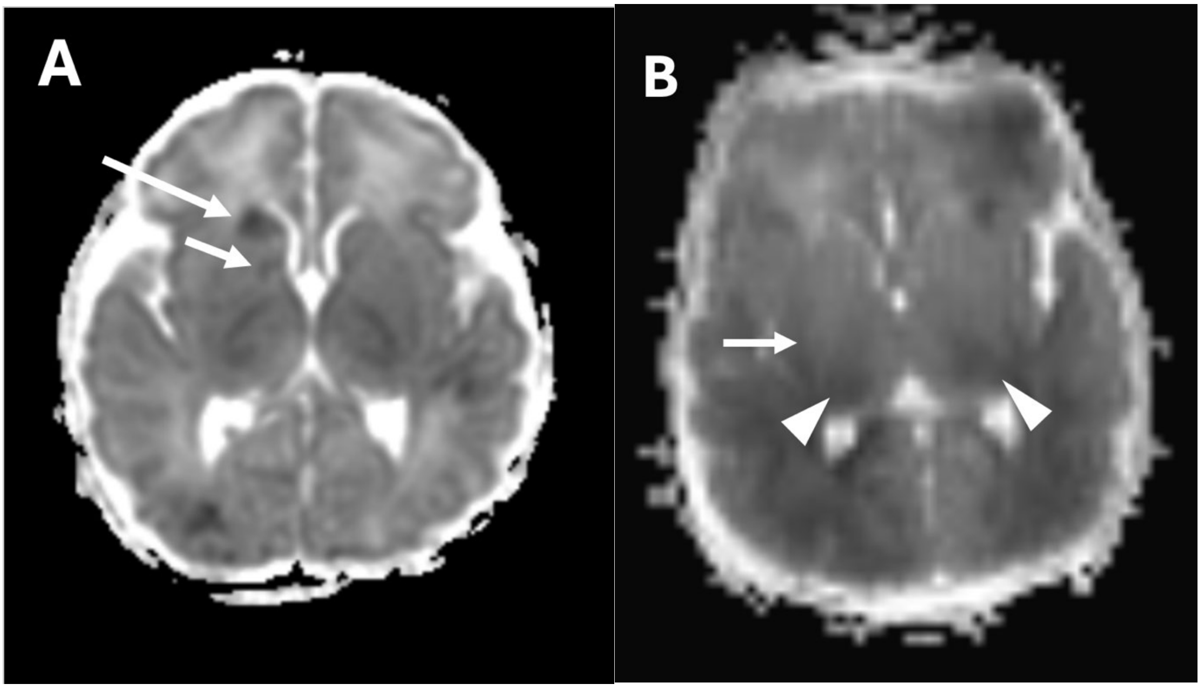

Supplement: Supplement 1. — eTable 1. Variables Considered for Inclusion in the 24h and Postcooling Models eTable 2. Outcome at Age 2 Years for All Surviving Infants Who Were Predicted to Have Death or Severe NDI for the 24h Model and the Postcooling Model eTable 3. Sensitivity Analyses to Evaluate Test Metrics for the Postcooling Model Based on Timing of MRI (Within or After 7 Days After Birth), Ignoring the 24 Hours Model (ie, Including All Neonates Who Survived to the End of Cooling), Using Only the Available MRI Data Without Imputation, and Including Only the Infants That Survived to Hospital Discharge eFigure 1. HEAL Trial Candidate Prediction Variable Selection Flow Diagram eFigure 2. Imaging From a False Positive MRI Results [file jamanetwopen-e2449188-s001.pdf]
