# Supplementary material for: Predictors of Death or Severe Impairment in Neonates With Hypoxic-Ischemic Encephalopathy
Source: JAMA Netw Open. 2024 Dec 5;7(12):e2449188. doi: 10.1001/jamanetworkopen.2024.49188 (PMC11621987; doi:10.1001/jamanetworkopen.2024.49188)
Supplement: Supplement 2. — Data Sharing Statement [file jamanetwopen-e2449188-s002.pdf]

Data Sharing Statement

Glass. Predictors of Death or Severe Impairment in Neonates with Hypoxic-Ischemic Encephalopathy. *JAMA Netw Open*. Published December 05, 2024.
doi:10.1001/jamanetworkopen.2024.49188

Data
Data available: Yes
Data types: Deidentified participant data
How to access data: The final study data set is accessible via a supervised private data enclave managed by the National Institute of Neurological Disorder and Stroke (NINDS) at: https://www.ninds.nih.gov/Current-Research/Research-Funded-NINDS/Clinical-Research/Archived-Clinical-Research-Data sets.
When available: beginning date: 01-01-2026

Supporting Documents
Document types: None

Additional Information
Who can access the data: The final study data set is accessible via a supervised private data enclave managed by the National Institute of Neurological Disorder and Stroke (NINDS) at: https://www.ninds.nih.gov/Current-Research/Research-Funded-NINDS/Clinical-Research/Archived-Clinical-Research-Data sets.
Types of analyses: The final study data set is accessible via a supervised private data enclave managed by the National Institute of Neurological Disorder and Stroke (NINDS) at: https://www.ninds.nih.gov/Current-Research/Research-Funded-NINDS/Clinical-Research/Archived-Clinical-Research-Data sets.
Mechanisms of data availability: The final study data set is accessible via a supervised private data enclave managed by the National Institute of Neurological Disorder and Stroke (NINDS) at: https://www.ninds.nih.gov/Current-Research/Research-Funded-NINDS/Clinical-Research/Archived-Clinical-Research-Data sets.
Any additional restrictions: The final study data set is accessible via a supervised private data enclave managed by the National Institute of Neurological Disorder and Stroke (NINDS) at: https://www.ninds.nih.gov/Current-Research/Research-Funded-NINDS/Clinical-Research/Archived-Clinical-Research-Data sets.
